# The epidemiology of pelvic ring fractures in Qatar

**DOI:** 10.1007/s00264-024-06103-w

**Published:** 2024-02-01

**Authors:** Mohamed H. Elamin, Islam Elkaramany, Loay A. Salman, Anas Albasha, Ashik Parambathkandi, Ahmed Elramadi, Ghalib Ahmed

**Affiliations:** 1 Orthopedics Department, Hamad General Hospital, Hamad Medical Corporation, PO Box 3050, Doha, Qatar; 2 Department of Orthopaedic Surgery, Surgical Specialty Center, Hamad Medical Corporation, Doha, Qatar

**Keywords:** Pelvic ring, Fractures, Epidemiology, Qatar

## Abstract

**Purpose:**

This study aimed to determine the incidence of pelvic ring fractures and their associated epidemiological profile in Qatar.

**Methods:**

A retrospective cross-sectional study was performed at the only level I trauma centre in Qatar for patients diagnosed with pelvic ring fractures between January 2016 and December 2018. Age, sex, mechanism of injury, fracture classification and associated characteristics, mode of treatment, associated nerve injuries, and other complications were analyzed.

**Results:**

A total of 327 consecutive patients were included, with an average age of 32.6 years. Most of the included patients were males, 85% (279), with a male: female ratio of 6:1. The incidence of pelvic fractures was 3.887/100,000 across the three years. High-speed motor vehicle collisions (MVC) were the most common mechanism of injury (108, 33%), followed by falling from height (105, 32%). Young-Burgess lateral compression (LC) fracture type was the most frequent (224, 68.5%) and was associated with 25% of the entire mortalities. Nine (2.8%) cases were open fractures, and 12% (39) were deemed unstable. Around 29% of cases had associated injuries, with an overall mortality rate of 4.9% (16) observed. Most fractures were treated nonoperatively(*n* = 283,86.5%).

**Conclusion:**

This study demonstrated the epidemiology of pelvic fractures in Qatar. MVC and work-related injuries were predominant in a younger cohort compared to the literature. Also, the mortality rate was lower than those reported in the literature. Therefore, well-trained surgeons and specialized trauma centres for treating these injuries are recommended.

## Introduction

Pelvic ring fractures account for around 2–8% of all skeletal fractures and can reach up to 25% in polytrauma patients [1–3]. They often result from high-energy trauma (i.e. motor vehicle collision (MVC) and falling from height (FFH)) in young patients and trivial falls in the elderly [1, 3]. Pelvic fractures are commonly associated with other skeletal and extra-skeletal systematic injuries; thus, thorough and urgent assessment with a multidisciplinary approach is warranted for better outcomes [4].

The mortality rate associated with pelvic fractures varies based on the type of fracture, especially open fractures [5], and the presence of associated injuries [6, 7]. Furthermore, the approach to managing pelvic ring fractures has undergone significant changes over the years [8]. There has been a shift from a more conservative approach in the past to surgical fixation and early mobilization, leading to notable improvements in the outcomes of pelvic ring fractures [2].

Numerous studies have reported the epidemiology of pelvic ring injuries in diverse populations worldwide, with an incidence rate of 20 to 34.30 per 100,000 [1, 2, 9–13]. Qatar, being one of the fastest-developing economies globally, experiences a substantial economic and healthcare burden [14]. It serves a unique demographic, with 80% of the population comprising young male expatriate workers. In a previous multicentric study, traumatic pelvic fractures accounted for approximately 11% of trauma admissions in Qatar, with a relatively younger mean age of 32 ± 14 and male predominance of 88% [15].

This study aimed to further investigate the primary characteristics of pelvic ring injuries, associated complications, and treatment trends among the young population of Qatar over a three-year period.

## Methods and materials

A retrospective, cross-sectional analysis was conducted following institutional approval by the review board at Hamad General Hospital (HGH), which is the country's sole level-one trauma centre. HGH serves a 2.7 million population and holds accreditation from the Joint Commission International (JCI) and the Accreditation Council of Graduate Medical Education-International (ACGME-I).

### Data source and collection

Electronic medical records were reviewed retrospectively for patients with pelvic ring fractures treated at HGH between January 2016 and December 2018. All patients with pelvic ring injuries were included in the study. Only patients with insufficient documentation and imaging studies were excluded. Two authors performed the search and data collection independently. Conflicts were resolved via a discrepancy meeting with a third, more senior pelvic surgeon.

Patients' demographics were logged into an electronic data spreadsheet. The baseline variables collected included mechanism of injury, neurovascular injuries, haemodynamic and fracture stability, treatment methods (operative vs conservative), complications, mortality, hospital stay, ICU admissions, association with other injuries, including acetabulum fractures, spine, head, chest and abdominal injuries.

Using Young and Burgess classification [16], pelvic fractures were classified into lateral compression type (LC), anteroposterior compression (APC), or vertical shear (VS). LC injuries are subdivided into LC1, LC2, and LC3. Similarly, APC injuries are subdivided into APC1, APC2 and APC3. Fractures were classified by senior surgeons using radiographs and CT scan images.

### Statistical analysis

For statistical analysis, categorical and continuous data were expressed in percentage, mean, and range and were correlated using percentages and graphical comparisons. Descriptive statistics were used to summarize the demographic, classification, treatment methods, and complications. All statistical analyses were performed using the statistical packages IBM SPSS Statistics for Windows, Version 25.0. (Armonk, NY: IBM Corp.) [17]. No power analysis was performed because all patients who met the inclusion criteria were included in this study.

## Results

A total of 327 patients who fulfilled the inclusion criteria with a mean age of 32.6 years were included in the study. Patients were primarily males, 85% (279), and 15% (48) females. The overall incidence of pelvic fractures was 3.887/100,000 across the three years period. A summary of study characteristics shown in Table 1.

**Table 1:** Demographic data

Total number	327
Gender	
Male	279 (85.3%)
Female	48 (14.7%)
Average age	32.6 (range 1–78)
Age groups (years)	
< 18	45 (13.8%)
19–30	111 (33.9%)
31–50	140 (42.8%)
51–70	26 (8.0%)
> 70	5 (1.5%)
Overall Incidence	3.887/100,000

### Incidence over time

From 2016 to 2018, the overall incidence of pelvic fractures fluctuated over three years. Based on an estimated Qatar population of 2,650,000, 2,720,000, and 2,780,000 in 2016, 2017, and 2018, respectively, the calculated annual incidence rate of pelvic ring injuries in Qatar was 2.113 per 100,000 in 2016 (65 cases), 5.735 per 100,000 in 2017 (156 cases), and 3.813 per 100,000 in 2018 (106 cases).

### Trends in age and gender

**Fig. 1:**
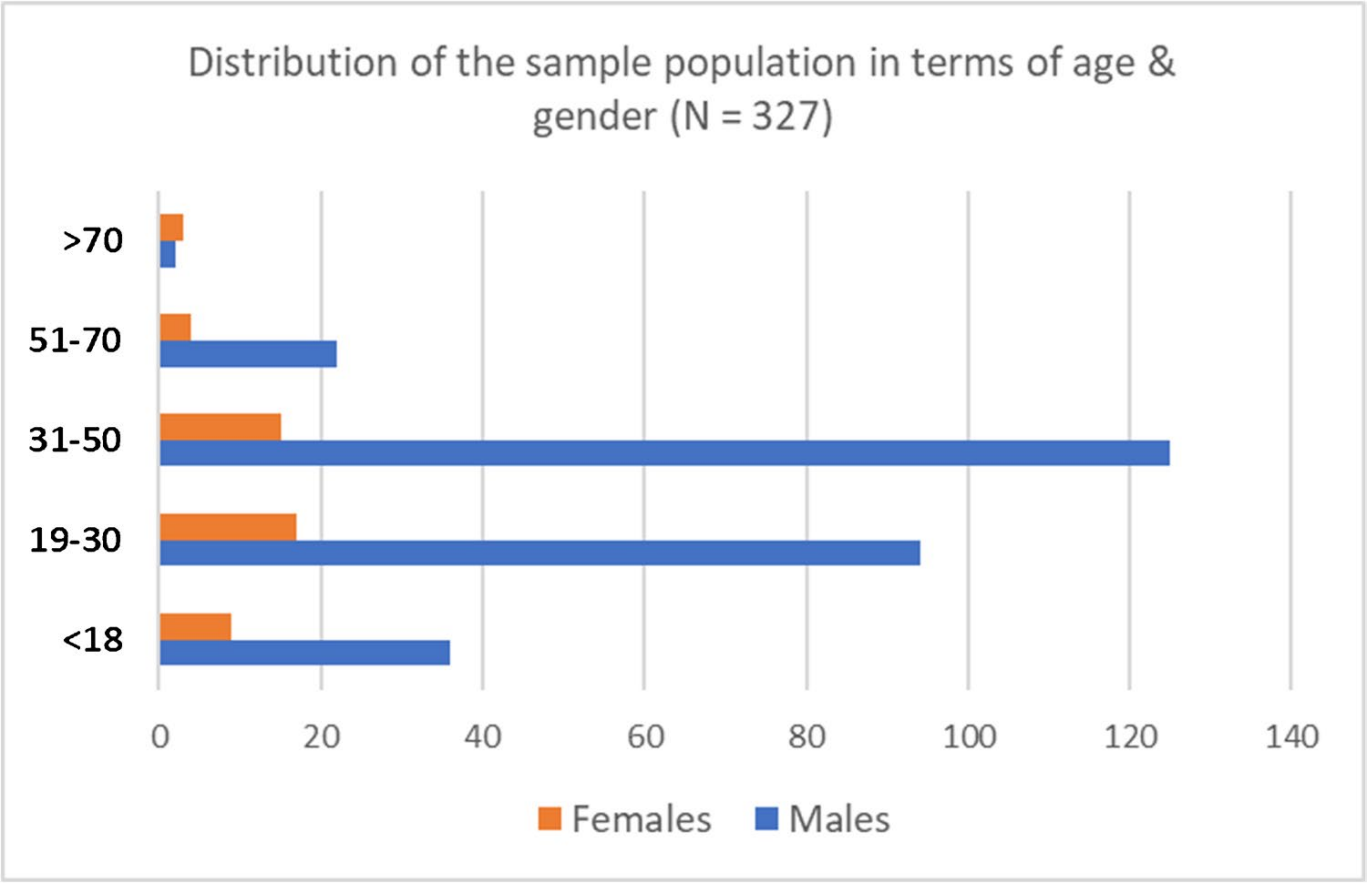
Pelvic ring fractures distribution according to age and gender

The mean age of patients was 32.6 years. Patients were divided according to age into five main groups, as illustrated in Table 1 and Fig. 1. Most patients (42.8%) fell into the 31–50 age category, followed by the 19–30 years group. Forty-five (13.8%) patients were of pediatric age (< 18 years). The incidence per age group reduced as the age increased above 50. A male to female ration of 6:1 was observed (Fig. 1).

### Fractures classification and characteristics

**Fig. 2:**
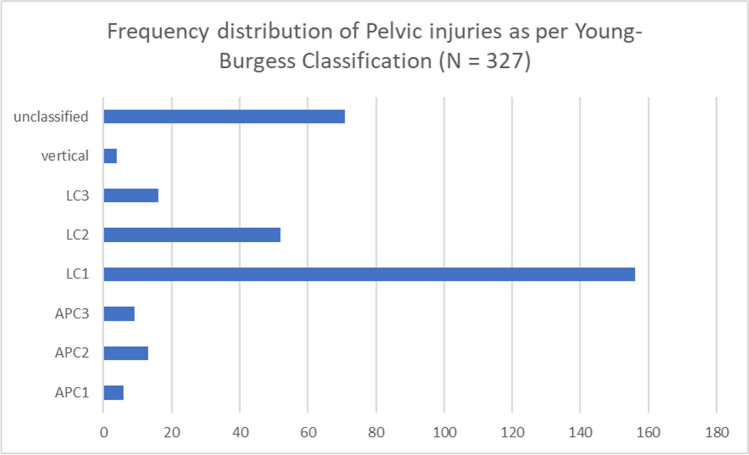
Frequency distribution of pelvic ring injuries according to Young-Burgess classification. APC: Anterior posterior compression type. LC: Lateral compression type

**Fig. 3:**
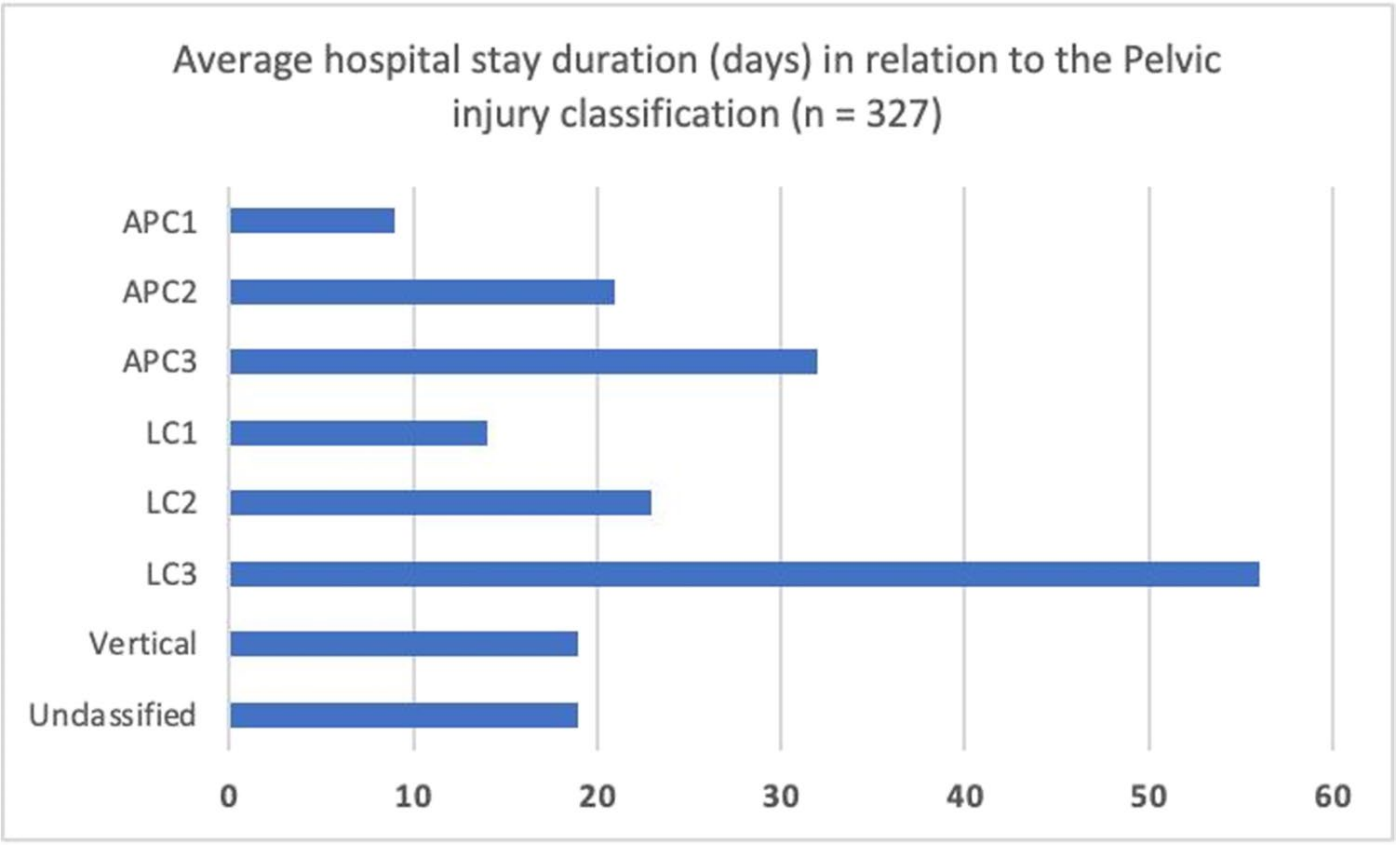
The average hospital stay based on YB classification. APC: Anterior posterior compression type. LC: Lateral compression type

While lateral compression (LC) fracture patterns were the most predominant, with 224 cases of hospitalized pelvic injuries (68.5%) and 25% deaths, vertical sheer type (VC) was the least observed fracture type (5%) (Fig. 2). Additionally, 5% (16) of inpatient mortalities were of lateral compression type. Furthermore, the average hospital stay across all Young-Burgess types was 24 days, with LC 3 type having the longest period of 56 days and APC 1 type having the least with an average of 9 days (Fig. 3)**.** Of the 327 fractures, 2.8% (9 cases) were open, and 1.5% (5 cases) were associated with neurological deficits.

In terms of patient and fracture stability, 261 (79.8%) of the fractures were pelvic and haemodynamically stable; however, 45 cases (13.8%), 39(11.9%), and 19(5.8%) were associated with either haemodynamic instability, or pelvic instability, or combined pelvic and haemodynamic instability, respectively. In addition, arterial injuries were reported in 30 cases (9.2%).

### Mechanism of injury

**Fig. 4:**
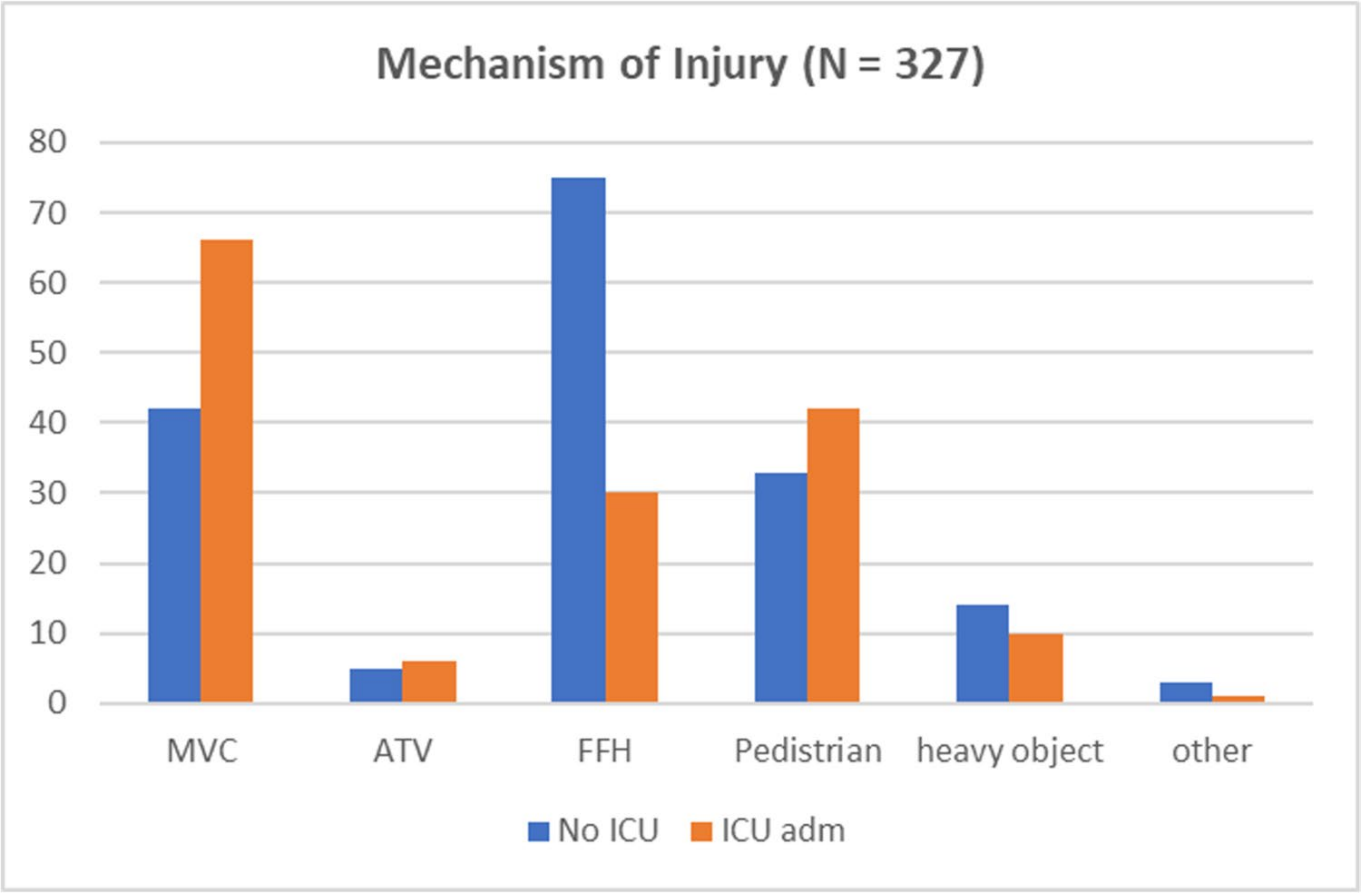
Mechanism of injury and its association with ICU admission. MVC: Motor vehicle collision, ATV: All-Terrain Vehicle, FFH: Fall from height

High-speed Motor Vehicle collisions (MVC) were the most common mechanism of injury related to pelvic fractures, contributing to around 33% (108) of the cases and the highest ICU admissions with 66 patients (42.5%). Fall from height (FFH) was the second leading cause of pelvic fractures, with 105 cases, followed by Pedestrians hit by a vehicle (75 cases; 23%) which was the most serious in terms of mortalities, with the highest inpatient death rate of 56.3% (9 patients) (Fig. 4).

### Associated Injuries

**Fig. 5:**
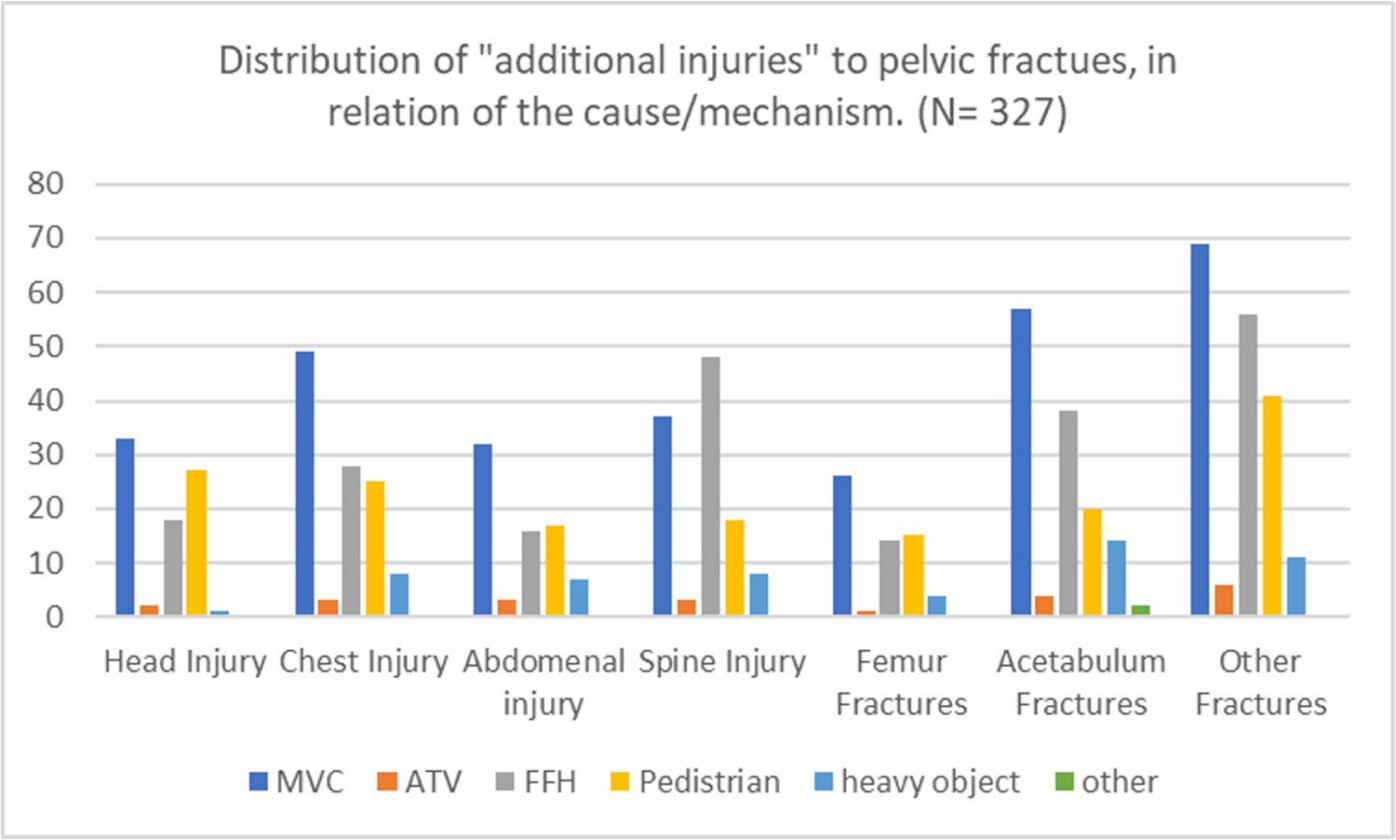
Distribution of other injuries in addition to pelvic fractures in relation to mechanism of injury. MVC: Motor vehicle collision, ATV: All-Terrain Vehicle, FFH: Fall from height

**Fig. 6:**
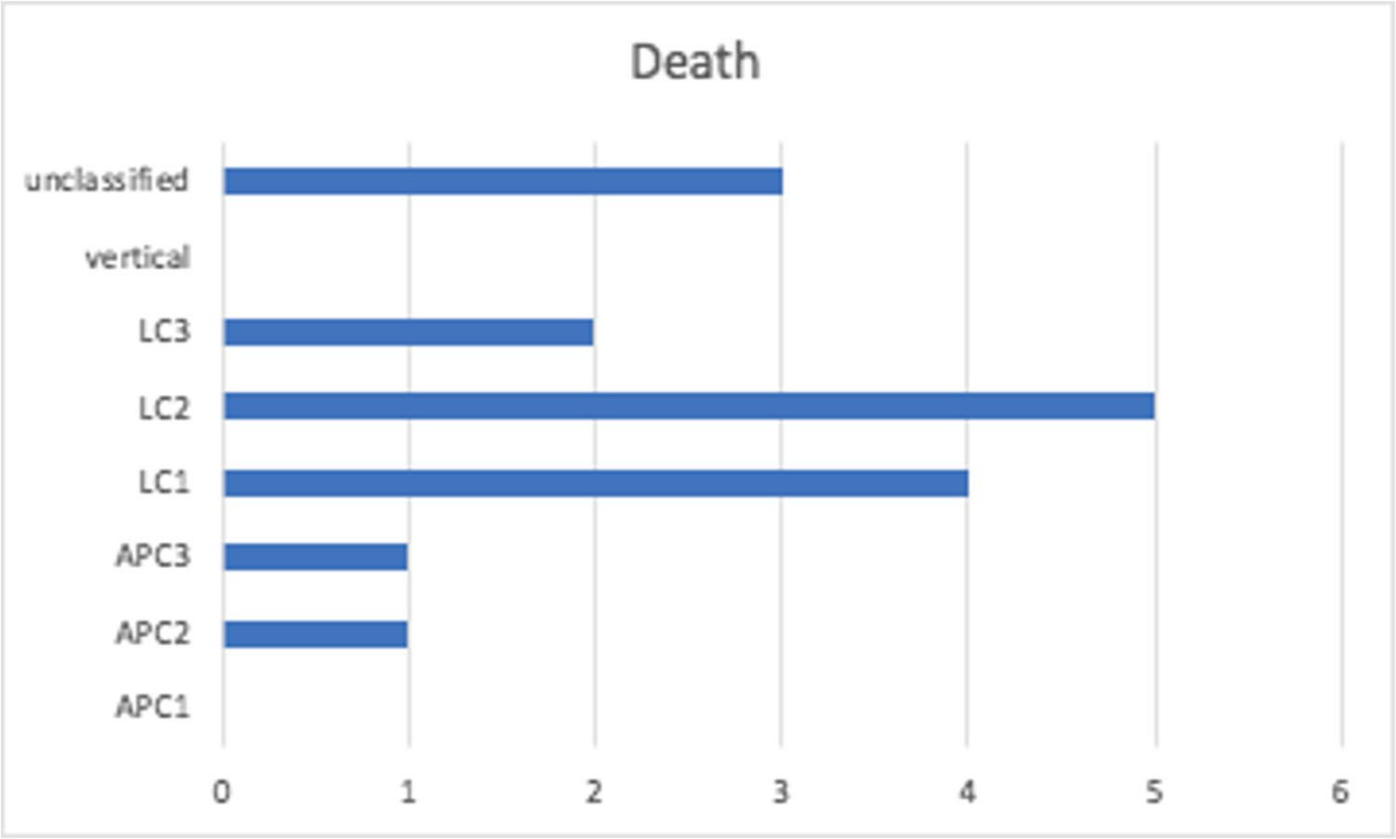
Mortality relation to Young-Burgess classification. APC: Anterior posterior compression type. LC: Lateral compression type

In the distribution of other injuries with the mechanism of injury, Acetabulum fracture was the highest (57 cases) in relation to MVC followed with second leading cause FFH (38 cases), followed by chest injuries (48 cases) with MVC then FFH (28 cases), in contrast to spinal injuries with the highest case is FFH (48 cases) followed by MVC (37 cases) (Figs. 5 and 6).

## Discussion

This study aimed to report the epidemiology of pelvic ring fractures in the state of Qatar over three years, from 2016 to 2018, at HGH, the sole trauma centre in the country. Pelvic ring fractures are perhaps the most severe and life-threatening orthopaedic injuries; therefore, appropriate early management is critical. With high mortality rates, pelvic ring injuries account for approximately 10% and 13% of all blunt trauma admissions in the US and Germany, respectively [15, 18] This high mortality is mainly attributed to the haemodynamic instability resulting from haemorrhage.

Compared to the 34.30 per 100.000 US incidence rate, the overall incidence rate of pelvic fractures in Qatar was 3.887/100,000, a significantly lower rate than in the US. Also, the incidence trend over the three years varied from 2016 to 2018 (2.113 per 100,000 in 2016, 5.735 per 100,000 in 2017, and 3.813 per 100,000 in 2018), a fluctuation that can be attributed to the rapid increase in infrastructure construction projects prior to the 2022 world cup and the subsequent strict application of labours rights legislations introduced by the local government [19].

Pelvic ring fractures account for around 2–8% of all skeletal fractures and can reach up to 25% in polytrauma patients [1, 2, 8], with inconsistent mortality based on fracture type, particularly in open pelvic fractures [9] and the presence of other associated injuries [4, 5]. In line with the current literature, this study has shown that LC fracture type contributed to nearly 68.5% of all hospitalized pelvic injuries and 25% of deaths.

Owing to the younger population, the mean age of pelvic fracture patients in Qatar was 32.6 years, compared to 48 years old in Europe [1]. In contrast to western cohorts, where female predominant distribution is observed (69.7%) [2, 15], 85% of pelvic fractures in Qatar were primarily reported in males, along with an unequal distribution across gender and a male: female ratio of about 6:1. This can be explained by the considerable male to female gap in the entire Qatar population [20].

In terms of modes of injury, this study has shown similar results compared to the literature [21, 22], in which the majority of fractures were caused by MVC (33%), followed by FFH (32%) and Pedestrians (23%), respectively.

Pelvic ring fractures are often associated with severe complications. In this cohort, arterial injuries were reported in 9.2% of the cases, mainly the internal iliac artery, with findings comparable to a study published by Pereira et al. [23]. In addition, 13.8% had haemodynamic instability, 11.9% had pelvic instability, 5.8% had both pelvic and haemodynamic instability, and 5% cases had died during their hospitalization in comparison to international data that showed mortality risk between 5–45% in all pelvic fracture [24–26]. This low mortality rate of 5% can reflect either good hospital care or poor management at the injury scene.

A much longer average hospital stay of 24 days was observed in our population compared to the US of 6.5 days in the US; however, a subanalysis of hospital stays based on the fracture classification showed similar trends in both countries with longer stays in LC3 type.

To the best of our knowledge, no previous studies have investigated the epidemiology of pelvic ring fractures in Qatar. However, several limitations must be acknowledged, including the inevitable selection bias resulting from the retrospective nature of this study and the potential for survival bias by excluding cases of death at the scene and on arrival. Furthermore, a longer follow-up period would have allowed for better observation of long-term outcomes and complications.

## Conclusion

This study has revealed a distinct epidemiological pattern of pelvic fractures in Qatar, characterized by an incidence rate of 3.887 per 100,000. Also, there is a higher prevalence of male and younger patients with pelvic fractures in Qatar, in contrast to the higher occurrence of female and older patients with pelvic fractures in Western nations. This cohort not only contributed valuable national data but may also help facilitate international comparisons.

## Data Availability

Happy to provide access to data (coding) upon request.
